# Is patient discharge after blood culture collection in the emergency department safe? A retrospective study in Japan

**DOI:** 10.1017/ash.2022.80

**Published:** 2022-05-16

**Authors:** Toshiki Miwa, Akane Takamatsu, Hitoshi Honda

## Abstract

**Background:** Drawing blood cultures in the emergency room (ER) is essential for detecting bloodstream infections (BSIs). Although a practice of drawing blood culture usually indicates a presence of severe infection requiring hospitalization, some patients may nonetheless be safely discharged from the ER. Previous studies demonstrated that patients with a positive blood culture after ER discharge had favorable clinical outcomes. Moreover, given the increasing incidence of febrile illnesses, especially in the era of COVID-19, the shortage of inpatient hospital beds may lend further justification to this practice. We investigated the prevalence, outcomes, and factors associated with patient discharge from the ER after blood collection. **Method:** The present, nested, case–control study comparing patients initially discharged from the ER with those directly admitted to the study institution was conducted at a 790-bed tertiary-care medical center in Tokyo, Japan. The ratio of the respective patients was 1:3. Factors associated with ER discharge after a blood-culture collection were identified using multivariate logistic regression analysis. **Results:** From January 2014 through December 2020, 153,432 patients visited the ER. Blood cultures were obtained for 19,010 patients; 2,575 (13.5%) of these had a true BSI, and of the latter, 142 (5.5%) were initially discharged from the ER. During 2020, the proportion of patients with ER discharge increased 1.7 times over previous years. There was no significant difference in 28-day mortality between the groups (2.1% vs 4.5%; *P* = .31). On multivariate logistic regression analysis, factors significantly associated with the decision to discharge after blood culture collection were the absence of hypotension (aOR], 14,92; 95% CI, 3.38–65.93), lack of altered mental status (aOR, 8.44; 95% CI, 3.28–21.71) at ER presentation, unknown diagnosis at ER discharge (aOR, 3.75; 95% CI, 1.97–7.16), high level C-reactive protein (aOR, 0.91; 95% CI, 0.87–0.94), and a diagnosis of intra-abdominal or hepatobiliary infection (aOR, 0.11; 95% CI, 0.04–0.29). **Conclusions:** ER discharge after drawing blood for a culture was more frequently seen in the current COVID-19 era and was deemed acceptable under certain circumstances, such as patients with no systemic illnesses or specific diagnosis who may be managed safely without compromising clinical outcomes.

**Funding:** None

**Disclosures:** None

